# A complicated case of expanded dengue syndrome presenting with dengue hemorrhagic fever in a five year old

**DOI:** 10.12669/pjms.41.8.11688

**Published:** 2025-08

**Authors:** Hina Naveed, Tehreem Afzal, Amna Masood, Abdul Salam Wazir

**Affiliations:** 1 Hina Naveed Department of Paediatric Medicine, Federal Government Polyclinic Hospital, Islamabad 44000, Pakistan; 2 Tehreem Afzal Department of Paediatric Medicine, Federal Government Polyclinic Hospital, Islamabad 44000, Pakistan; 3 Amna Masood Department of Paediatric Medicine, Federal Government Polyclinic Hospital, Islamabad 44000, Pakistan; 4 Abdul Salam Wazir Department of Paediatric Medicine, Federal Government Polyclinic Hospital, Islamabad 44000, Pakistan

**Keywords:** Dengue fever, Encephalitis, Expanded dengue syndrome, Myocarditis

## Abstract

Dengue fever is an endemic disease in Pakistan presenting with a spectrum of clinical manifestations, ranging from mild fever to severe complications such as life-threatening bleeding and even death. Expanded dengue syndrome is a less recognized and rarely reported manifestation of the viral illness. We report a complicated case of expanded dengue syndrome in a five-year-old female initially presenting with Dengue Hemorrhagic Fever and subsequently involving multiple systems during the course of illness. She developed acute acalculous cholecystitis, hand cellulitis, right pleural effusion, encephalitis and ultimately myocarditis during her sixteen days of hospital admission. Despite the severity of her condition she was discharged in a vitally stable condition. This case highlights the importance of thorough monitoring and clinical vigilance to ensure timely recognition and management of such rare presentations in dengue fever patients in order to overcome this diagnostic challenge.

## INTRODUCTION

Dengue fever is an arboviral illness endemic in tropical and subtropical climates with highest prevalence in Asia.^1^ It usually presents with biphasic high-grade fever, severe myalgia, headache with retro-orbital pain and vomiting. The dengue virus is transmitted through the bite of the female Aedes aegypti, a daytime biting mosquito that predominantly breed in freshwater in highly urbanized localities. The viral illness exists in a spectrum ranging from fever to disordered hemostasis and capillary leakage. World Health Organization (WHO) reclassified dengue in 2009 which included probable dengue, laboratory confirmed dengue with or without warning signs and severe dengue.^2^

In 2012, WHO further expanded the literature to include the phenomenon called Expanded Dengue Syndrome (EDS), an umbrella term used to refer to unusual isolated or multisystem presentations of dengue.^3^ EDS includes but is not limited to cardiovascular, renal, pulmonary, hepatic and central nervous system. Though, EDS is uncommonly recognized and reported, it is imperative to timely identify such complications to reduce morbidity and mortality associated with severe dengue infection. Hence, to add to the meagre body of literature, we report a case of EDS in a five-year-old girl that presented to Federal Government Polyclinic Hospital in Islamabad Pakistan with dengue hemorrhagic fever having subsequent cardiac, CNS and GI involvement.

## CASE PRESENTATION

We report a five year old female child who presented with fever for the last five days, mild epistaxis, fresh blood in stool and intermittent severe abdominal pain. On examination her heart rate was 95 beats/min, respiratory rate was 26 breaths/min, temperature 99F, blood pressure 100/60mmHg. There was no active bleed from any site. Further examination revealed tenderness in right upper quadrant of abdomen. Laboratory investigations showed Dengue IgM positive, Hb 9.8g/dl, Hct 27.7, TLC 81x10^3^/ UL (83.2% neutrophil), platelet count 52x10^3^/UL. BSR, serum electrolytes, creatinine were normal. Suspicion of abdominal compartment syndrome was made. USG abdomen revealed thick edematous gall bladder wall of 4.9mm thickness with normal pancreas and hepatic echotexture and no ascites or stone.

Hence the diagnosis of *Acute Acalculous Cholecystitis* was made and the child was managed conservatively with judicious use of IV fluid and injection ceftriaxone for prevention of secondary bacterial infection. Patient entered the critical phase of disease on second day of admission. The lowest recorded platelet count was 32x10^3^/UL on third day of admission after which they increased gradually to be in the normal range. On fifth day of admission she developed right hand *cellulitis*. The hand up to wrist was warm, erythematous, tender, swollen with restriction of movement but palpable radial pulse. She was shifted to injection meropenem, started on injectable linezolid for seven days with fusidic acid cream for local application.

**Fig.1:**
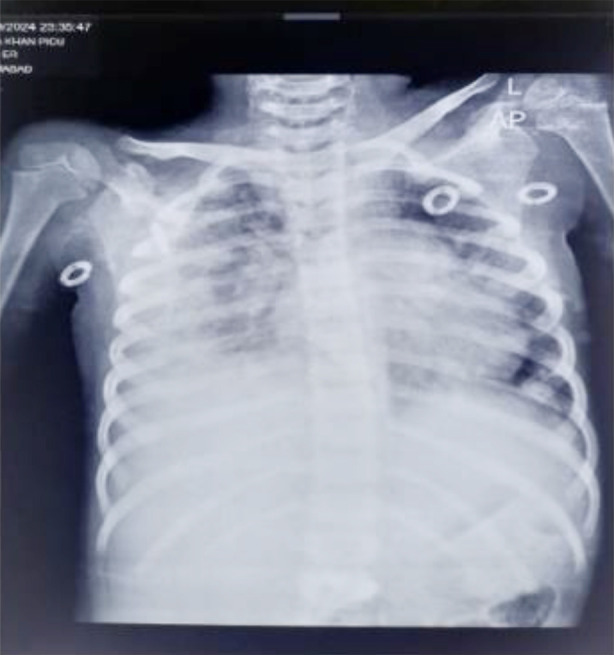
CXR showing right sided pleural effusion.

On the same day she started experiencing breathing difficulty. Examination revealed increased distress, decreased air entry on right lung base with dull percussion note and decreased vocal resonance. Her oxygen saturations were deteriorating so she was shifted to manual bubble CPAP @ 4L O_2_/min. CXR had obliteration of right costophrenic angle as shown in Fig.1. Ultrasound chest revealed right mild free fluid in pleural cavity (257ml) and diagnosis of *Right Pleural Effusion* was made. The serum albumin level was 2.1g/dl. She was given nebulization of ipratopium bromide and beclomethasone. Two doses of injectable furosemide were given. Gradually she was weaned off oxygen with improved breathing and decrease in pleural fluid (112ml) as evidenced by repeat ultrasound chest done on ninth day of admission.

On seventh day of admission she had altered level of consciousness with intermittent abnormal jaw movements. EEG showed encephalopathy consistent with *dengue encephalitis*. CSF RE was also consistent with viral encephalitis. However, dengue antibodies could not be done due to unavailability in our setup. On eight day of admission she had a vague complain of chest pain. Precordial auscultation revealed a gallop rhythm. Cardiac enzymes were raised. LDH 408 U/L (normal range 60-170U/L), D-dimers 512 ng/ml (normal range <200ng/ml), serum ferritin 537 ng/ml (normal range 7-140ng/ml).

**Fig.2:**
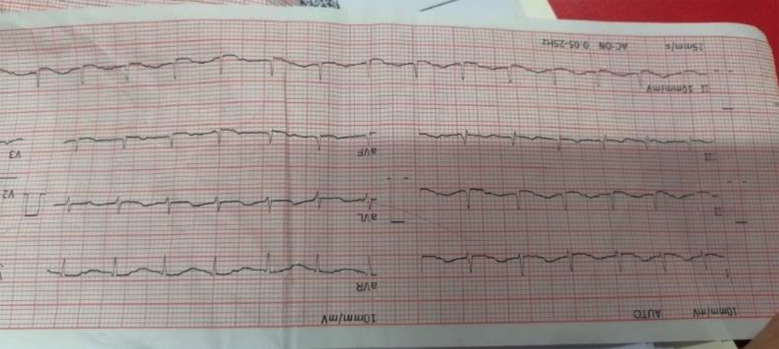
ECG showing low-voltage with suspicion of myocarditis.

ECG showed low voltage QRS complexes as shown in Fig.2. Echocardiography revealed ejection fraction of 45%. Diagnosis of *dengue myocarditis* was made. She was started on tablet spironolactone, tablet enalapril and tablet digoxin. ECHO was repeated after five days with improvement in ejection fraction to 55%. She continued tablet enalapril for one month. Patient was discharged on sixteenth day of admission. She was active, vitally stable, with no breathing difficulty, normal conscious level, orally tolerating well and pain-free.

## DISCUSSION

Expanded dengue syndrome includes unusual presentations of dengue fever like neurological, cardiac, renal and liver abnormalities.^4^ In the endemic areas atypical symptoms are increasingly identified. In this case report, the patient had dengue fever along with cholecystitis, cellulitis, encephalitis and myocarditis. To our best of knowledge, this has not been reported in our region. Initially our patient had acute acalculous cholecystitis which was managed conservatively. Cholecystistis in dengue fever is due to edema of gall bladder wall due to increased vascular permeability.^5^ Setwayati reported that an 11 years old female with abdominal pain was diagnosed on ultrasound abdomen similar to our patient.^6^

Our patient had cellulitis of the hand up to the arm on day five. Several cases of orbital cellulitis attributed to viral replication and direct infection of the skin by the virus have been reported in dengue fever^7^, however cellulitis of the arm has not been reported before. Cellulitis of the hand may have the same mechanism. Our patient subsequently developed right pleural effusion which was managed conservatively. Capillary leakage into the lungs causing effusion is well established in dengue shock syndrome. Various authors have reported multiple cases.^8^ On seventh day patient was diagnosed with encephalitis. Neurological manifestations having incidence upto 5.4% in 225 dengue patients studied in Pakistan include altered level of consciousness (58.3%), seizures (41.6%), neck stiffness (16.6%), decerebration (16.6%) and hemiplegia/paresis (33.3%).^9^ Our patient had altered level of consciousness and abnormal movements of jaw. CSF RE was consistent with viral encephalitis. Lastly our patient had myocarditis with an ejection fraction of 45%. This could be due to direct virus attack on the myocytes, capillary leakage or intracellular calcium homeostasis alteration. Dengue myocarditis has been reported by multiple authors in pediatric patients having symptoms similar to our patient.^10^

### Limitations:

This case report has a few limitations. CSF antibody test for DENV igM was unavailable in our setup. Further case studies should be carried out in pediatric age group to gain more evidence of unusual manifestations of dengue fever.

## CONCLUSION

Dengue in endemic areas may present with many atypical features. Multiple system involvement can occur at the same time at the time of presentation or it can involve different organs in its course of illness. This case report explains that early diagnosis, timely intervention and close monitoring of dengue fever can be a life saving measure for children. The paediatricians should have a high index of suspicion of these rare presentations of an otherwise common infectious disease especially in endemic areas.

### Authors’ Contribution:

**HN:** Conceived, designed, collected data, patient assessment & edited manuscript, is responsible for integrity of research. **HN, TA and AM:** Literature search and manuscript writing. **ASW:** Did critical review and final approval of manuscript.
